# Stereochemistry of Consabatine from *Convolvulus sabatius* Viv. (Convolvulaceae)

**DOI:** 10.3797/scipharm.1208-14

**Published:** 2013-02-05

**Authors:** Sonja Christina Ott, Kristina Jenett-Siems, Eckart Eich

**Affiliations:** Institut für Pharmazie (Pharmazeutische Biologie), Freie Universität Berlin, Königin-Luise-Str. 2-4, D-14195 Berlin, Germany.

**Keywords:** *Convolvulus sabatius*, Convolvulaceae, Consabatine, 3α-Tropanol ester, Mosher esters

## Abstract

The stereochemistry of consabatine, which was isolated from the roots of *Convolvulus sabatius* Viv. as a novel natural compound, has now been determined by the synthesis of its Mosher esters. Consabatine was found to be 1′*R*-configurated.

## Introduction

Tropane alkaloids are one of the most important and widespread groups of secondary metabolites in the Convolvulaceae. From the roots of the Mediterranean *Convolvulus sabatius* Viv., consabatine was isolated as a new natural compound [1]. This extraordinary 3α-tropanol ester comprises an unusual isoprenylated cyclohexenylic acid as its acylic component. Mainly from the *Merremia* species, several related aromatic substances called merresectines – esters of 3α-tropanol with kurameric acid/nervogenic acid and derivatives – have been isolated as well [2]. Especially from the chemotaxonomic point of view, the identification of consabatine and the related merresectines is of significance, as they are specific to Convolvulaceae so far.

## Results and Discussion

**Fig. 1:**
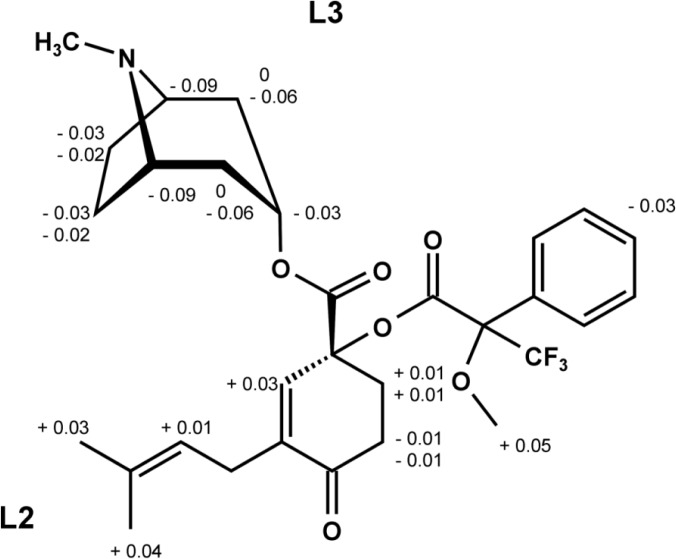
MTPA-consabatine with differences Δδ (*S* – *R*) taken from the ^1^H-NMR spectra of *S*-MTPA- and *R*-MTPA-consabatine

To clearly define a natural compound, the knowledge of not only its molecular structure, but also of its stereochemistry is essential. In order to determine the absolute configuration of C-1′ in the terpenoid moiety of consabatine, the advanced Mosher method was applied [3]. After preparation of the epimeric Mosher esters, *S*-MTPA-consabatine and *R*-MTPA-consabatine, they were submitted for ^1^H-NMR spectroscopy. As described in [4], the differences in the protons’ chemical shifts Δδ (*S* – *R*) between *S*-MTPA- and *R*-MTPA-consabatine were calculated (Fig. 1). According to [3], negative Δδ-values point to an orientation above the MTPA plane (L3), and positive values to an orientation below the MTPA plane (L2). As a consequence, consabatine shows a 1′*R*-configuration.

## Experimental

### General procedures

^1^H-NMR and ^1^H-^1^H-COSY spectra were obtained on a Bruker AMX 400 MHz (TMS as internal standard). The EIMS was recorded on a Varian MAT 711 (70 eV).

### Plant material

Several specimen of *Convolvulus sabatius* Viv. were bought at Gartencenter Pluta, Berlin. They were cultivated and harvested at the Berlin Botanical Garden.

### Extraction and isolation of consabatine

The dried and ground roots of *Convolvulus sabatius* were extracted with methanol three times. After evaporation of the solvent, the residue was dissolved in 2% aqueous tartaric acid and extracted with petrolether, dichloromethane, and ethyl acetate. Then, the aqueous layer was alkalinized (pH 10) with 25% aqueous NH_3_ and extracted with dichloromethane again. This alkaloidal extract was separated by means of preparative HPLC (0.5% aqueous H_3_PO_4_/MeOH 80:20 to 40:60 in 60 min) and preparative TLC (CHCl_3_/MeOH/aq. NH_3_conc. 80:20:2). Consabatine was verified by ^1^H-NMR and EIMS measurements.

Consabatine (12.2 mg), (1*R*,3*r*,5*S*)-8-Methyl-8-azabicyclo[3.2.1]octan-3-yl (1*R*)-1-hydroxy-3-(3-methylbut-2-en-1-yl)-4-oxocyclohex-2-ene-1-carboxylate: ^1^H-NMR (400 MHz, CDCl_3_): δ 6.40 (1H, s, H-2′), 5.10 (2H, t, *J* = 5.0 Hz, H-3/H-2″), 3.14 (2H, br s, H-1/H-5), 2.96 (2H, br d, *J* = 7.0 Hz, CH_2_-1″), 2.74 (1H, ddd, *J* = 5.5 Hz, 7.5 Hz, and 17.1 Hz, H-5′d), 2.62 (1H, ddd, *J* = 5.2 Hz, 8.9 Hz, and 17.1 Hz, H-5′u), 2.37 (1H, tt, *J* = 5.6 Hz, and 7.5 Hz, H-6′d), 2.30 (3H, s, N–CH_3_), 2.24 (1H, dt, *J* = 5.3 Hz, and 8.7 Hz, H-6′u), 2.20 (2H, m, H-2ax/H-4ax), 2.02 (2H, m, H-6exo/H-7exo), 1.73 (3H, s, CH_3_-4″), 1.72 (2H, m, H-2eq/H-4eq), 1.70 (2H, d, *J* = 8.1 Hz, H-6endo/H-7endo), 1.60 (3H, s, CH_3_-5″); EIMS (70 eV): *m/z* (rel. int.) 347 (16), 330 (1), 223 (1), 141 (7), 140 (3), 125 (12), 124 (100), 97 (9), 96 (18), 95 (8), 94 (8), 83 (26), 82 (21).

### Synthesis of the Mosher esters of consabatine

One-half of the consabatine obtained (6.1 mg) was dissolved in 0.5 mL anhydrous dichloromethane. Then 8.8 mg dimethylaminopyridine (DMAP), 3.7 μL triethylamine (TEA), and 6.6 μL (–)-α-methoxy-α-(trifluoromethyl)phenylacetic acid (MTPA) chloride were added under nitrogen atmosphere. The mixture was stirred overnight. To terminate the reaction, 4.34 μL 3-[(dimethylamino)propyl]amine (3-DMAPA) was added, and the mixture was stirred for 10 min. After evaporation of the solvent, the residue was applied to the preparative TLC (CHCl_3_/MeOH/aq. NH_3_ conc. 40:10:1) to give *S*-MTPA-consabatine.

*S*-MTPA-consabatine (4.2 mg): ^1^H-NMR (400 MHz, CDCl_3_): δ 7.36–7.45 (5H, m, aromatic protons), 6.92 (1H, s, H-2′), 5.22 (1H, t, *J* = 4.4 Hz, H-3), 5.06 (1H, br t, *J* = 7.0 Hz, H-2″), 3.78 (1H, m, H-5′d), 3.76 (1H, m, H-5′u), 3.57 (3H, s, N–CH_3_), 3.55 (3H, s, O–CH_3_), 3.39 (2H, d, *J* = 7.3 Hz, H-2ax/H-4ax), 3.09 (2H, m, H-1/H-5), 2.95 (2H, br d, *J* = 7.4 Hz, CH_2_-1″), 2.47 (2H, m, H-6exo/H-7exo), 2.29 (1H, m, H-6′d), 2.24 (1H, m, H-6′u), 2.12 (2H, br d, *J* = 16.1 Hz, H-6endo/H-7endo), 1.92 (2H, br d, *J* = 16.6 Hz, H-2eq/H-4eq), 1.76 (3H, s, CH_3_-4″), 1.61 (3H, s, CH_3_-5″).

The second half of consabatine (6.1 mg likewise) was treated in the same manner with (+)-MTPA chloride instead to give *R*-MTPA-consabatine.

*R*-MTPA-consabatine (3.7 mg): ^1^H-NMR (400 MHz, CDCl_3_): δ 7.38–7.49 (5H, m, aromatic protons), 6.89 (1H, s, H-2′), 5.25 (1H, t, *J* = 5.1 Hz, H-3), 5.05 (1H, br t, *J* = 7.3 Hz, H-2″), 3.78 (1H, m, H-5′d), 3.75 (1H, m, H-5′u), 3.54 (3H, s, N–CH_3_), 3.50 (3H, s, O–CH_3_), 3.39 (2H, d, *J* = 7.2 Hz, H-2ax/H-4ax), 3.18 (2H, m, H-1/H-5), 2.96 (2H, br d, *J* = 7.0 Hz, CH_2_-1″), 2.50 (2H, m, H-6exo/H-7exo), 2.30 (1H, m, H-6′d), 2.25 (1H, m, H-6′u), 2.14 (2H, br d, *J* = 15.8 Hz, H-6endo/H-7endo), 1.98 (2H, br d, *J* = 16.4 Hz, H-2eq/H-4eq), 1.73 (3H, s, CH_3_-4″), 1.57 (3H, s, CH_3_-5″).
